# Body shape trajectories are associated with birth weight, body mass index and sociodemographic conditions in participants of the Brazilian Longitudinal Study of Adult Health (ELSA-Brasil): a multiple correspondence analysis

**DOI:** 10.1186/s12889-023-16779-1

**Published:** 2023-09-25

**Authors:** Isiyara Taverna Pimenta, Rosane Harter Griep, Sheila Maria Alvim de Matos, Maria de Fatima Haueisen Sander Diniz, Arlinda B. Moreno, Maria de Jesus Mendes da Fonseca

**Affiliations:** 1 National School of Public Health, Oswaldo Cruz Foundation, Rio de Janeiro, Brazil; 2 Laboratory of Health and Environment Education, Oswaldo Cruz Institute, Oswaldo Cruz Foundation, Rio de Janeiro, Brazil; 3 Institute of Collective Health, Federal University of Bahia, Salvador, Brazil; 4 Brazilian Longitudinal Study of Adult Health, Federal University of Minas Gerais, Belo Horizonte, Brazil

**Keywords:** Body shape, Body weight changes, Body weight trajectory, Birth weight, Sociodemographic factors

## Abstract

**Background:**

Evaluating lifelong weight trajectories is challenging due to the high costs of studies that follow individuals from childhood to adulthood. The use of silhouette scales has been a new approach to assess the body shape trajectory across life as a proxy for body weight trajectory. Depending on body shape trajectories, individuals may be more prone to develop diseases in adulthood. Therefore, identifying factors related to them is essential for public health. This study aimed to evaluate body shape trajectories across the lifespan and to verify associations between them, birth weight, body mass index, and sociodemographic conditions in a Brazilian cohort.

**Methods:**

This is a cross-sectional analysis conducted with 14,014 participants of first follow-up data collection of Longitudinal Study of Adult Health (ELSA-Brasil). ELSA-Brasil is a multicentric prospective cohort study initiated in 2008 with civil servants of six public institutions in the Northeast, South and Southeast regions of Brazil. We applied a clustering method to longitudinal data to identify body shape trajectories from 5 to 40 years of age and assessed the associations between these trajectories and birth weight, body mass index and sociodemographic conditions (race, education, maternal education and monthly per capita family income) using multiple correspondence analysis.

**Results:**

We found five body shape trajectories for women and three for men. Low birth weight was associated with a slight to moderate increase in shape. High birth weight was associated with maintaining large body size in both sexes and markedly increased body shape in women. Higher sociodemographic status and white race were associated with marked increases in body shape in men and maintenance of medium body shape in women.

**Conclusions:**

The study shows that variables related to worse lifetime weight status (evaluated by anthropometry), such as presence of obesity, are also associated with worse body shape trajectories, as assessed with silhouette scales. Our results suggest that body shape trajectories are a good indicator of body weight trajectories and may be used when cohort studies are not possible.

**Supplementary Information:**

The online version contains supplementary material available at 10.1186/s12889-023-16779-1.

## Background

Body weight trajectory is defined as changes in weight over life, and one way to evaluate it is the use of silhouette scales, a set of figures representing the body, from very lean to very heavy [1]. Using the silhouettes chosen by individuals to represent their bodies at previous ages and using appropriate analysis (like cluster methods for longitudinal data), it is possible to determine self-perceived body shape trajectory [2–5]. The latter can be used as a proxy for body weight trajectory because silhouettes scales have high correlation with body mass index (BMI) and they work well to identify obesity and underweight in the Brazilian adult and older adult population [6, 7].

Sociodemographic variables are important for explaining weight changes. Low educational level can negatively influence the opportunities for good employment and contribute to low family income. In this scenario, racial inequalities play a major role in Brazil, since blacks Brazilian earn lower average incomes than whites, which can be explained by lower schooling, occupation of less prestigious job positions and racial discrimination [8]. Meanwhile, low family income affects place of residence, offering less access to safe and suitable locations for leisure-time physical exercise and healthy food outlets, as well as limiting the family funds available for food [9–11]. Physical activity and healthy eating are well-established strategies for individual weight control and obesity prevention [12, 13].

Early life is a critical period for development. Some exposures in the first 1,000 days (from conception to two years of age) impact child development and can favour excess weight [14]. In this context, low maternal education is a major factor related to insufficient prenatal care and excess gestational weight gain [15, 16], which in turn affect foetal and infant health. Women with more schooling are presumably more likely to care for themselves, have more knowledge about selfcare and enjoy higher socioeconomic status [15]. As described above, poor socioeconomic conditions can limit access to nutritionally balanced food, including during pregnancy, which affects the infant´s birth weight. Birth weight is an indicator of nutritional sufficiency during gestation and is related to the child´s subsequent weight status. Studies have observed that higher birth weight (≥ 4,001 g) is associated with higher odds of excess weight in childhood [17] and that low birth weight (≤ 2,500 g) is associated with increased odds of childhood obesity and underweight [18]. Children with obesity are also more likely to present pre-obesity or obesity in adulthood [19, 20].

Individuals with body shape trajectories characterized by a marked increase in weight or maintenance of large body size across their lifespan may be more prone to develop chronic diseases in adulthood or older age [3–5]. In this context, identifying factors related to these trajectories is essential for encouraging the development of preventive public health strategies. Until this moment, we have not identified any studies that have evaluated body weight trajectories using silhouette scales to identify changes in weight within a Brazilian population. Additionally, we have not found any studies conducted with Brazilian and other populations that have assessed factors associated with body shape trajectories. The current study thus aimed to evaluate body shape trajectories from 5 to 40 years of age and verify associations between them and birth weight, current BMI, and sociodemographic conditions in participants in the Longitudinal Study of Adult Health (ELSA-Brasil).

The present study will contribute to the current scientific literature on body shape trajectories by utilizing a novel method to assess changes in weight across the lifespan within a Brazilian population. This method enables the evaluation of weight changes even in the absence of cohort studies. In addition, this study has the potential to encourage the development of public policies aimed at promoting changes in modifiable factors associated with body shape trajectories that are related to negative health outcomes.

## Methods

### Study population

ELSA-Brasil is a multicentre cohort study that began in 2008–2010 with 15,105 civil servants from 35 to 74 years of age, affiliated with six public institutions in the Northeast, South and Southeast regions of Brazil. Participants answered face-to-face questionnaires on sociodemographic aspects, health-related habits and behaviours, medical history and mental health and underwent clinical examinations. Additional information on this cohort is available in a previous publication [21].

**Fig. 1:**
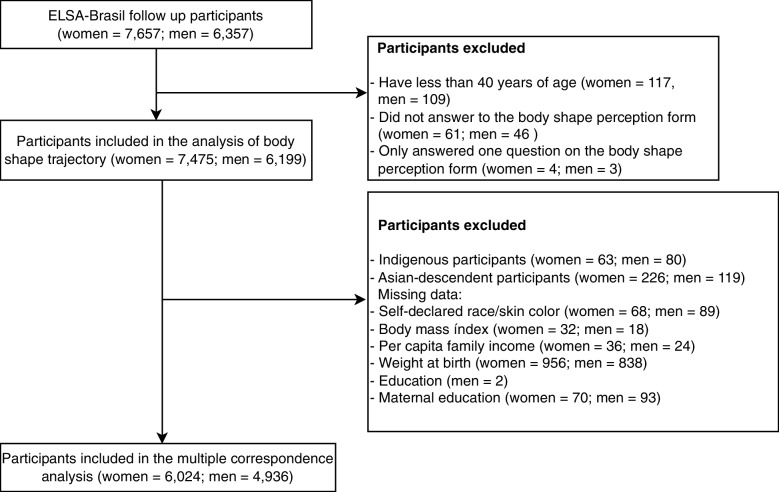
Flowchart of participants, ELSA-Brasil, first follow up wave data (2012–2014)

The current study is a cross-sectional analysis that used data from the first follow-up wave of ELSA-Brasil, which was conducted 3.8 years after the baseline with 14,014 participants, 7,657 women (54,6%) and 6,357 men (45,4%) at 38 to 79 years of age (retention rate of 93%). We excluded participants with age less than 40 years (women = 117, men = 109) because the selected scale for body shape trajectories follows participants until this, those who failed to answer the body shape scale (women = 61, men = 46) and those who only answered one question (women = 4, men = 3), leaving a total of 13,674 individuals (women = 7,475; men = 6,199). In the multiple correspondence analysis, we excluded participants that declared their race as indigenous or Asian-descendant due to the small numbers of participants in these categories (1% and 2.5%, respectively) and those with missing data for any of the study variables, resulting a total of 10,960 participants (6,024 women and 4,936 men) (Fig. 1).

### Body shape trajectory assessment

Participants were asked to identify their body shapes at 5, 10, 20, 30 and 40 years of age by choosing one of the nine silhouettes developed by Stunkard, Sorensen and Schulsinger [1] in the first follow up wave. The instrument's validity for measuring past shapes was assessed by Must, Willett and Dietz and it was considered satisfactory [22]. For quality control, we assessed the stability (using the test–retest method) of the silhouettes chosen by a sample consisting of 204 participants who responded to the body shape perception form administered during the first follow-up of the ELSA-Brasil study. The sample size was determined for all research centres of the ELSA-Brasil study, with a significance level (alpha error) of 5%. Quotas were established based on age categories (38–47, 48–57, 58–67, and 67 years or older) and occupational categories (support, high school, and university) to ensure that the selected sample was representative of the entire cohort population. An interval of 7 to 14 days was adopted between the test and the retest. The stability was considered good and very good, based on the cut-off points established by Byrt [23], with values ranging from 0.68 to 0.79 for women and 0.63 to 0.83 for men, considering all the body shapes selected in the test and retest (Additional file 1).

### Birth weight, BMI and sociodemographic conditions

Birth weight was self-reported by participants with the following question: “According to the information you have, what was your birth weight? Below 2.5 kg, 2.5 kg to 4 kg or over 4 kg?". Birth weight less than 2.5 kg was defined as low, from 2.5 to 4 kg as adequate and greater than 4 kg as high.

To determine BMI weight was measured with a digital scale (Toledo®, São Bernardo do Campo, São Paulo, Brazil) accurate to 50 g, and height (m) was measured with a fixed stadiometer (Seca®, Hamburg, Germany) accurate to 0.1 cm, according to the protocol proposed by Lohman, Roche and Martorell [24]. BMI cut-off points were < 24.9 kg/m^2^ for underweight and normal range (these categories were grouped because less than 1% of the participants were underweight), 24.9 to 29.9 kg/m^2^ for pre-obesity and ≥ 30 kg/m^2^ for obesity [12].

Sociodemographic variables were age (in years), sex (male and female), self-reported race (white, brown and black), participant´s education (up to secondary school and university), maternal education (up to elementary school and complete secondary school/university) and monthly per capita family income (low, middle and high). The income categories were defined based on terciles using the 2013 exchange rate to convert Brazilian reais to US dollars. The values for the low-income set at up to $795 for women and up to $675 for men, the middle-income range was defined as $795 to $1,446 for women and $675 to $1,374 for men, while the high-income category included amounts higher than $1,446 for women and higher than $1,374 for men.

We selected the birth weight, race, and maternal education variables because they precede body shape trajectories and may potentially explain their development, as they are associated with weight status [15, 25, 26]. We also chose participants’ education and per capita family income because, despite being variables related to the current stage of life, they are social determinants that influence health behaviors associated with weight status, such as diet and physical activity [26]. Additionally, we included the current BMI in the analyses because if the BMI categories align with body shape at 40 years, it will corroborate with silhouette scales are a suitable tool for evaluating body shape trajectories in a Brazilian population.

### Statistical analysis

We employed a clustering method for longitudinal data to identify body shape trajectories from 5 to 40 years of age. Cluster analysis is a statistical technique employed to create distinct and non-overlapping subgroups of individuals or objects, based on shared characteristics [27]. The cluster method used in this study (*kmlshape*) is based on a variation of the k-means algorithms and group trajectories according to their shapes using a distance measure that respects the form (generalized Fréchet distance) and a mean that respects the form (Fréchet average) [2]. Data simplification was used because the study population was large. The algorithm summarizes the total population in a smaller group of individuals when a data simplification procedure is used. The trajectories of the smaller groups are called “senators” because they represent the entire population. Senators are selected via classical k-means [2]. We tested 100 and 200 senators, because when we attempted to increase this number, some iterations in the analyses failed to converge. The results were similar using both senators’ numbers, so we present the results using 100 senators in this study. After data simplification, the clustering method chose the centres of the clusters by random selection and allocated participants to one of the trajectories for which they showed similar shapes. Subsequently, the average trajectories were plotted.

The trajectories were named according to their depiction, with silhouettes 1–2 defined as lean, 3–4 as medium shape, and 5–9 as heavy [28]. Theoretically, the nine potential body shape trajectories are stable shape (stable lean, stable medium, stable heavy), increasing body shape (lean-increasing, medium-increasing, heavy-increasing), and decreasing body shape (lean-decreasing, medium-decreasing, heavy-decreasing). We thus tested clusters with three to nine trajectories. We defined the number of body shape trajectories that best represented males and females using the generalized Fréchet distance matrix between the respective trajectory clusters and selected the number of clusters with the highest mean generalized Fréchet distance.

The associations between body shape trajectory and birth weight, maternal education, per capita household income, race, participant´s education and BMI were assessed by multiple correspondence analysis (MCA) and these variables were described using absolute and relative frequencies. MCA is an exploratory technique utilized to assess relationships or correspondence between categories of qualitative variables. This analysis visually illustrates the relationship between a set of variables, where the proximity of categories in space indicates a correspondence between them [29]. One advantageous aspect of MCA is that it does not assume any assumption concerning probability distributions, thus allowing the investigation of different patterns of association, including non-linear. The analysis provides total inertia, and the resulting square root (eigenvalue) corresponds to the total variance explained by each dimension [30]. The number of dimensions was chosen by analysing the decline in eigenvalues using scree plot. Dendrograms were performed (graphic representations of hierarchical cluster analysis) with the coordinates obtained in the MCA to determine the clusters, providing clear visualization of the categories of variables in each group [31]. Scatterplots were formed by the coordinates of each category in each dimension, and clusters of categories were used to identify factors associated with body shape trajectories. The scatterplot´s x-axis and y-axis represent the data´s variability explained by the first and second dimensions. The dots represent each variable category. The groups of associated variables categories were delineated by lines.

A previous study with participants in ELSA-Brasil found that body image differed between men and women. Men tended to underestimate and not distort their body size, while women tended to overestimate their body size [32]. The way individuals perceive their bodies can impact self-perceived body shape trajectories, so we stratified the analyses by sex. We attempted to stratify the analysis of body shape trajectories by age group (younger versus older adults), but since the trajectories were similar between the two age groups, we ultimately decided only to stratify the analyses by sex. The R software, version 4.0.5 [33], and the libraries “kmlshape,” “ca,” “factoextra,” “dendextend” and “ggplot2” were used. We set the significance level at 5%.

## Results

### Body shape trajectory

According to the highest mean of generalized Fréchet distance (Table 1), the number of trajectories from 5 to 40 years of age was three for men and four or five for women. Four versus five body shape trajectories for women produced similar results, so we chose five trajectories to represent the female population.

**Table 1:** Mean of the generalized Fréchet distance among the trajectories

Trajectory number	Mean of the generalized Fréchet distance	
	Women	Men
3	19.70	26.77
4	22.47	21.23
5	22.46	19.40
6	19.58	21.45
7	21.09	21.71
8	14.52	16.81
9	20.76	18.65

According to this measure, the ideal number of body shape trajectories to represent the male population was three and four or five for the female population

**Fig. 2:**
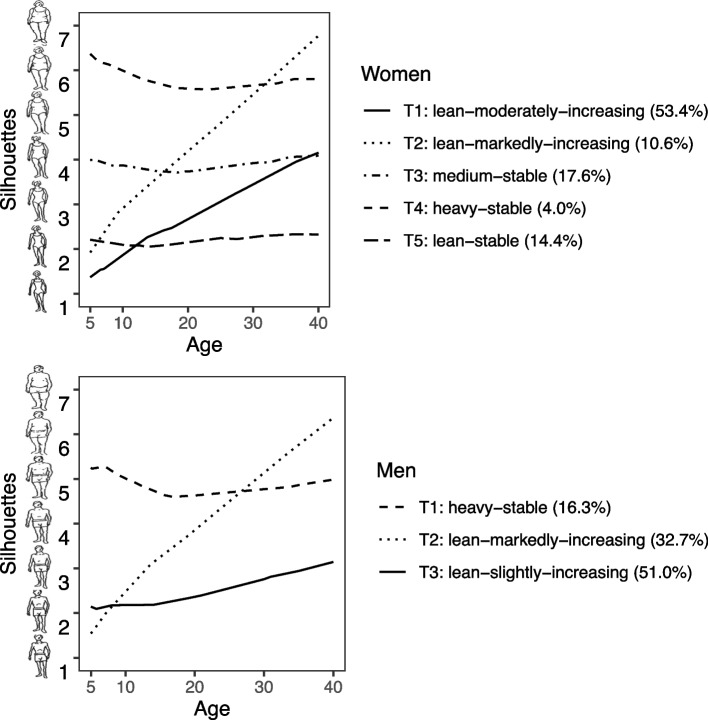
Body shape trajectories of women and men participating in the ELSA-Brasil. Note. We use longitudinal cluster data (kmlShape) to identify the trajectories

Approximately 11% of women and 33% of men had a lean-with-markedly-increasing body shape trajectory, in which they started lean and gained weight markedly until reaching a heavy body shape. Meanwhile, 4% of women and 16% of men showed a heavy-stable trajectory, characterized by maintenance of heavy body shape. In women, 18% showed a medium-stable trajectory, characterized by maintaining medium body shape throughout life, while 14% showed a lean-stable trajectory, marked by maintenance of lean shape throughout life, and 53% showed a lean-moderately-increasing trajectory, in which they started lean and gained weight until reaching medium body shape. Most men (51%) showed a lean-slightly-increasing trajectory, in which they began lean at five years of age and gained weight until reaching medium shape (Fig. 2).

### Multiple correspondence analysis

In the population included in multiple correspondence analysis mean age was 55 years for women (SD:8.3) and men (SD: 8.7). Most of the participants reported their race as white (women: 56.3%, men: 57%) and maternal schooling as primary or less (women: 75.5%, men: 73.8%). The majority of both sexes had university degrees (women: 62.5%, men: 56.7%), were pre-obese (women: 36.9%, men:47%), and reported adequate birth weight (women: 85.8%, men: 83%) (Table 2).

**Table 2:** Distribution of study variables from 10,960 participants included in multiple correspondence analysis, ELSA-Brasil, 2012–2014

Variables	Women (6,024) n (%)	Men (4,936) n (%)
**Age (mean** ± **SD)**	55.12 ± 8.32	55.13 ± 8.73
**Race**		
White	3,392 (56.3)	2,814 (57.0)
Brown	1,575 (26.1)	1,482 (30.0)
Black	1,057 (17.5)	640 (13.0)
**Maternal education**		
Primary or less	4,546 (75.5)	3,642 (73.8)
Complete secondary or university	1,478 (24.5)	1,294 (26.2)
**Education**		
Secondary or less	2,262 (38.5)	2,138 (43.3)
University	3,762 (62.5)	2,798 (56.7)
**Per capita family income**		
Low	1,938 (32.2)	1,604 (32.5)
Middle	2,045 (33.9)	1,844 (37.4)
High	2,041 (33.9)	1,488 (30.1)
**Body mass index**		
Normal	1,970 (32.7)	1,383 (28.0)
Pre obesity	2,223 (36.9)	2,319 (47.0)
Obesity	1,831 (30.4)	1,234 (25.0)
**Birth weight**		
Low	503 (8.3)	374 (7.6)
Adequate	5,170 (85.8)	4,098 (83.0)
High	351 (5.8)	464 (9.4)

*SD* Standard deviation

**Fig. 3:**
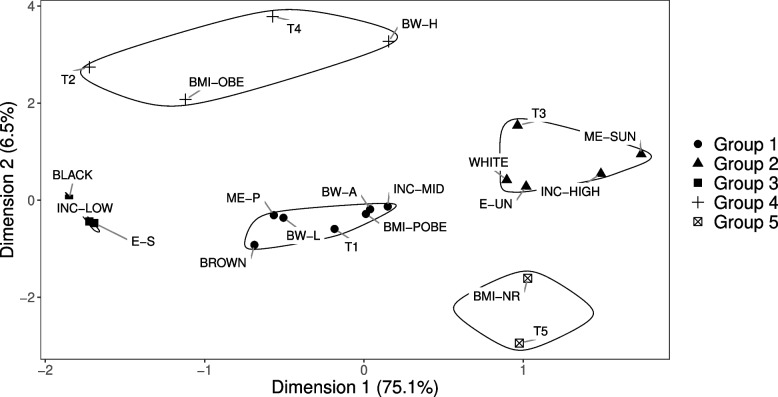
Two-dimensional plot of multiple correspondence in female participants in the ELSA-Brasil study (*n* = 6024). Note. T1 = lean-moderately-increasing trajectory; T2 = lean-markedly-increasing trajectory; T3 = medium-stable trajectory; T4 = heavy-stable trajectory; T5 = lean-stable trajectory; BW-L = low birth weight; BW-A = adequate birth weight; BW-H = high birth weight; ME-P = maternal education equivalent to primary school or less; ME-SUN = maternal education equivalent to complete secondary school or university; BMI-NR = normal body mass index; BMI-POBE = pre obesity; BMI-OBE = obesity; INC-LOW = low per capita family income; INC-MID = middle per capita family income; INC-HIGH = high per capita family income; WHITE = white race; BROWN = brown race; BLACK = black race; E-S = secondary education or less; E-UN = complete university education

The MCA plot allowed identification of five groups in women (Fig. 3), associations between variable categories and groups formation can be evaluated by analysing the proximity between points. The first dimension explained 75.1% of the data´s variability (x-axis) and the second explained 6.5% (y-axis). Considering the contributions of each variable category in the composition of each dimension, we observed that the second dimension was primarily formed by heavy-stable body shape trajectory, adequate and high birth weight. The other categories (except for the lean-stable trajectory and BMI obesity) were important in forming dimension one (Chart S1, Additional file 2).

Maternal education primary or less, adequate and low birth weight, brown race, middle per capita family income and pre-obesity were associated with lean-moderately-increasing body shape trajectory (Group 1). White race, maternal education equivalent to secondary school or university, high per capita family income and participant´s education equivalent to university were associated with the medium-stable trajectory (Group 2). Obesity and high birth weight were associated with heavy-stable and lean-markedly-increasing trajectories (Group 4), and normal BMI was associated with the lean-stable trajectory (Group 5) (Fig. 3).

**Fig. 4:**
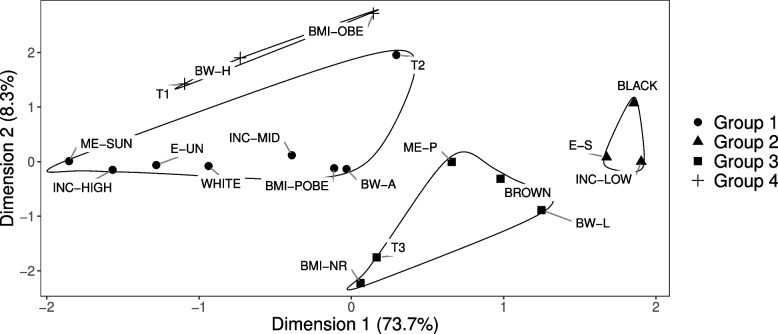
Two-dimensional plot of multiple correspondence in male participants in the ELSA-Brasil study (*n* = 4936). Note. T1 = heavy-stable trajectory; T2 = lean-markedly-increasing trajectory; T3 = lean-slightly-increasing trajectory; BW-L = low birth weight; BW-A = adequate birth weight; BW-H = high birth weight; ME-P = maternal education equivalent to primary school or less; ME-SUN = maternal education equivalent to complete secondary school or university; BMI-NR = normal body mass index; BMI-POBE = pre obesity; BMI-OBE = obesity; INC-LOW = low per capita family income; INC-MID = middle per capita family income; INC-HIGH = high per capita family income; WHITE = white race; BROWN = brown race; BLACK = black race; E-S = secondary education or less; E-UN = complete university education

The MCA plot allowed identification of four groups in men (Fig. 4). The first dimension explained 73.7% of the data´s variability (x-axis) and the second explained 8.3% (y-axis). The second dimension was primarily formed by lean-markedly-increasing and lean-slightly-increasing body shape trajectories, adequate birth weight, BMI normal range and obesity. The other categories were important in forming dimension one (Chart S2, Additional file 2).

White race, maternal education equivalent to complete secondary school or university, high and middle per capita family income, participant´s education equivalent to university, pre-obesity and adequate birth weight were associated with the lean-markedly-increasing body shape trajectory (Group 1). Maternal schooling primary or less, brown race, low birth weight and normal range BMI were associated with the lean-slightly-increasing trajectory (Group 3). Obesity and high birth weight were associated with the heavy-stable trajectory (Group 4). Two cluster groups (two for men and three for women) were not associated with any body shape trajectory, including participant´s secondary schooling or less, low per capita family income and black race (Fig. 4).

## Discussion

The study´s objective was to evaluate body shape trajectories from 5 to 40 years of age and verify associations between them and birth weight, current BMI, and sociodemographic conditions in participants in the ELSA-Brasil study. We found five body shape trajectories for women and three for men. Low birth weight was associated with a slight increase in shape among men and a moderate increase in shape among women. High birth weight was associated with worse body shape trajectories in women (heavy-stable and lean-markedly-increasing) and men (heavy-stable). Higher sociodemographic status and white race were associated with lean-markedly-increasing body shape trajectory in men and medium-stable trajectory in women.

A study conducted with 11,423 Spanish participants of both sexes from the Seguimiento Universidad de Navarra (SUN) cohort found five body shape trajectories from 5 to 40 years for men and women using the same silhouette scales employed in the present study. The analysis was not stratified by sex. The authors also found the lean-moderate increase and medium-stable trajectories, just like in the present study. However, they found trajectories such as medium-moderate increase, heavy-medium, and heavy-moderate increase, which differed from the trajectories found in the present study [28].

Low birth weight can indicate nutritional insufficiency during pregnancy and in the present study it was associated with lower maternal schooling, brown race and lean-moderately-increasing body shape trajectory in women and lean-slightly-increasing trajectory in men. For pregnant women to have access to quality food in sufficient quantity, they need favourable financial conditions. If the study participants' mothers have lower schooling, they probably tended to hold poorer paid jobs, impacting food´s availability and affordability during the pregnancy. Exposure to food deprivation during the pregnancy may result in subsequent accumulation of adipose tissue when food availability is restored, predisposing to obesity throughout life [34]. Thus, nutrient shortage during gestation may have promoted adaptive responses during foetal development, resulting in moderate and slight weight gain across women´s and men´s lives.

Previous studies found an association between high birth weight and excess weight at different moments in the life cycle, including childhood, adolescence and adulthood [35]. In the present study, high birth weight was also associated with maintenance of heavy body shape across life in both sexes and a marked weight increase across women´s lives. Since childhood and adolescence are critical periods of development, excess weight in these stages affects weight status in subsequent life stages, including increased likelihood of pre-obesity or obesity in adulthood [19, 20]. Individuals that maintained a heavy body shape and those with a marked increase in weight across life had higher risk of hypertension [3], excessive daytime sleepiness [36], vascular ageing [37], diabetes mellitus [38] and all-cause and cardiovascular mortality [4] when compared to those who maintained medium or lean body shape across life.

As expected, obesity was associated with a heavy-stable body shape trajectory in both men and women and a lean-markedly-increasing trajectory in women. Pre-obesity was associated with a lean-moderately-increasing trajectory in women and lean-markedly-increasing trajectory in men. Normal BMI was associated with a lean-stable trajectory in women and lean-slightly-increasing trajectory in men. The results reflect consistency between current weight status (assessed by BMI) and body shapes at 40 years and corroborate that the silhouette scale can be a useful tool for assessing body shape trajectories.

The highest categories of maternal education, per capita family income, and participant´s education, besides white race, were associated with the medium-stable trajectory in women. In contrast, among men, the same sociodemographic conditions were associated with poor body shape trajectory, characterized by a marked increase in body shape across life reaching obesity at 40 years old. These results can be explained by gender differences in eating patterns. The quantity and quality of food consumed is a gender marker. Studies show that women tend to eat more healthily than men, which includes higher consumption of fruits and vegetables, and lower consumption of fatty meats, meat products, and alcohol. In addition, women receive greater pressure from society to maintain a slim body, which can make them worry about their diet and practice physical activity for weight management and maintenance of adequate weight [39, 40].

Although black race, low per capita family income and secondary schooling or less were not associated with any body shape trajectory, these categories were associated with each other. This result corroborates findings in the Brazilian population (although ELSA-Brasil consisted of civil servants). Data published by the Brazilian Institute of Geography and Statistics (IBGE) [41] in 2020 showed racial segregation in the labour market before the COVID-19 pandemic. Most brown and black Brazilians worked at occupations that required less schooling and that provided lower income (e.g., construction, domestic work, agriculture). On the other hand, most white Brazilians worked in higher-paid occupations (e.g., public administration, teaching, health, social services) that required higher education.

The current study has some limitations. Body shape trajectories depend on the participants’ perception of their own bodies and are susceptible to distortions, which may overestimate or underestimate the body shape. Thus, the results may not represent participants’ true body weight trajectories. However, considering the high cost and difficulty of conducting longitudinal studies that follow individuals from childhood into adulthood, we believe the assessment of body shape trajectories can help elucidate the implications of weight changes across life. Multiple correspondence analysis represents the researcher’s view of specific issues, so other researchers may not consider the same variables, and different results can be obtained. The birth weight and maternal education information were self-reported, so there may have been a misclassification.

Despite the above-mentioned limitations, our study was the first to show the contribution by birth weight and sociodemographic variables to perceived weight changes over time. We stratified the analyses by sex considering that perceived body image is different for men and women, with men tending to underestimate and not distort their body size, while women tend to overestimate their body size [32]. Furthermore, the satisfactory stability of the body sizes chosen by the participants to indicate previous body sizes strengthens the results obtained in the trajectory analysis (Additional file 1) and contributes to expanding the assessment of the psychometric quality of the silhouette scales used. We use a clustering method for longitudinal data that respects the form of trajectories to group the individuals, this technique also allows defining the body shape trajectories of individuals who do not have all time points evaluated, avoiding many exclusions and thus the use of imputation methods [2].

Childhood and adolescence are critical periods of development as they involve the consolidation of lifestyle and behavioural patterns. The adoption of unhealthy habits and behaviours, often influenced by peers or family, can lead to nutritional inadequacies during this life stage and have long-term implications for health. Obese children and adolescents are at a higher risk of developing obesity in adulthood [42, 43]. So, we believe that these stages of life have a greater influence on the determination of body shape trajectories. Current literature has shown that exposure to adverse situations in childhood and adolescence, such as physical, emotional, verbal, and sexual abuse; household substance abuse; mental illness; domestic violence, emotional, psychological; parental separation or divorce; household criminality; neglect; bullying; and serious illness or injury, is associated with negative long-term health outcomes, such as overweight or obesity [44]. However, these conditions were not evaluated in the present study because these variables were not available in the ELSA-Brasil study. It is suggested that future research assess how these adverse experiences in childhood and adolescence can influence the development of different body shape trajectories across the lifespan. Additionally, as we have not identified studies that have assessed the association between body shape trajectories and health outcomes in the Brazilian population, such as the development of chronic diseases and mortality, we suggest that future studies investigate these associations. Understanding how different body shape trajectories are related to health outcomes can provide important information for the prevention and management of long-term health conditions.

## Conclusions

The variables described in the literature as associated with worse weight status (evaluated by BMI) in specific life moments were also associated with worse body shape trajectories (estimated using silhouette scales). In this scenario, we think silhouette scales can be used to define changes in weight across life, mainly when conducting cohort studies is not possible. Public policies that promote antenatal care and improvements in sociodemographic conditions can positively impact body shape trajectories.

### Supplementary Information

**Additional file 1:** **Table S1.** Stability (test-retest) of the silhouette scales chosen to represent body sizes from 5 to 40 years old, according to sex, in ELSA-Brasil participants (*n*=204).

**Additional file 2:** **Chart S1.** Distribution of the absolute and relative contribution of each variable category in the female correspondence analysis for the two dimensions. **Chart S2.** Distribution of the absolute and relative contribution of each variable category in the male correspondence analysis for the two dimensions.

## Data Availability

The datasets used and/or analyzed during the current study are available from the corresponding author on reasonable request.
