# Supplementary material for: Body shape trajectories are associated with birth weight, body mass index and sociodemographic conditions in participants of the Brazilian Longitudinal Study of Adult Health (ELSA-Brasil): a multiple correspondence analysis
Source: BMC Public Health. 2023 Sep 25;23:1857. doi: 10.1186/s12889-023-16779-1 (PMC10518926; doi:10.1186/s12889-023-16779-1)
Supplement: Supplementary file 1 — Additional file 1: Table S1. Stability (test-retest) of the silhouette scales chosen to represent body sizes from 5 to 40 years old, according to sex, in ELSA-Brasil participants (n=204). [file 12889_2023_16779_MOESM1_ESM.pdf]

Supplementary material 1 – Stability (test-retest)

Table S1. Stability (test-retest) of the silhouette scales chosen to represent body sizes from 5 to 40 years old, according to sex, in ELSA-Brasil participants (n=204)

Age (years)	Women (n=100)	Men (n=104)
5	0.79 (0.66-0.91)	0.83 (0.69-0.96)
10	0.83 (0.74-0.91)	0.81 (0.69-0.94)
20	0.68 (0.54-0.83)	0.76 (0.65-0.86)
30	0.73 (0.59-0.86)	0.73 (0.61-0.86)
40	0.68 (0.54-0.86)	0.63 (0.43-0.82)

Note: Weighted kappa and respective 95% confidence intervals (95%CI).
