# Supplementary material for: Body shape trajectories are associated with birth weight, body mass index and sociodemographic conditions in participants of the Brazilian Longitudinal Study of Adult Health (ELSA-Brasil): a multiple correspondence analysis
Source: BMC Public Health. 2023 Sep 25;23:1857. doi: 10.1186/s12889-023-16779-1 (PMC10518926; doi:10.1186/s12889-023-16779-1)
Supplement: Supplementary file 2 — Additional file 2: Chart S1. Distribution of the absolute and relative contribution of each variable category in the female correspondence analysis for the two dimensions. Chart S2. Distribution of the absolute and relative contribution of each variable category in the male correspondence analysis for the two dimensions. [file 12889_2023_16779_MOESM2_ESM.pdf]

Chart S1. Distribution of the absolute and relative contribution of each variable category in the **female** correspondence analysis for the two dimensions

| Variables | Variables categories | Contribuitions | | | |
|---|---|---|---|---|---|
| | | Dimension 1 | | Dimension 2 | |
| | | Absolute | Relative | Absolute | Relative |
| Body shape trajectories | T1. lean-moderately-increasing trajectory | 0.003 | 0.260 | 0.027 | 0.229 |
| | T2. Lean-markedly-increasing trajectory | **0.046** | **0.591** | 0.117 | 0.129 |
| | T3. Medium-stable trajectory | **0.024** | **0.820** | 0.060 | 0.180 |
| | T4. Heavy-stable trajectory | 0.002 | 0.115 | **0.085** | **0.432** |
| | T5. Lean-stable trajectory | 0.019 | 0.307 | 0.175 | 0.240 |
| Birth weight | Low birth weight | **0.003** | **0.761** | 0.002 | 0.033 |
| | Adequate birth weight | 0 | 0.253 | **0.004** | **0.509** |
| | High birth weight | 0 | 0.016 | **0.089** | **0.625** |
| Maternal education | Maternal education equivalent to primary school or less | **0.034** | **0.933** | 0.010 | 0.024 |
| | Maternal education equivalent to complete secondary school or university | **0.106** | **0.933** | 0.032 | 0.024 |
| BMI | Normal range | **0.049** | **0.586** | 0.121 | 0.123 |
| | Pre obesity | 0 | 0.001 | 0.004 | 0.038 |
| | Obesity | **0.054** | **0.567** | 0.187 | 0.167 |
| Per capita family income | Low per capita family income | **0.137** | **0.826** | 0.009 | 0.005 |
| | Middle per capita family income | **0.001** | **0.485** | 0.001 | 0.031 |
| | High per capita family income | **0.107** | **0.824** | 0.014 | 0.010 |
| Race | White race | **0.065** | **0.928** | 0.014 | 0.017 |
| | Brown race | **0.018** | **0.771** | 0.032 | 0.119 |
| | Black race | **0.086** | **0.954** | 0 | 0 |
| Education | Secondary education or less | **0.154** | **0.826** | 0.012 | 0.005 |
| | Complete university education | **0.092** | **0.826** | 0.007 | 0.005 |

Chart S2. Distribution of the absolute and relative contribution of each variable category in the **male** correspondence analysis for the two dimensions

| Variables | Variables categories | Contribuitions | | | |
|---|---|---|---|---|---|
| | | Dimension 1 | | Dimension 2 | |
| | | Absolute | Relative | Absolute | Relative |
| Body shape trajectories | T1. Heavy-stable trajectory | **0.028** | **0.745** | 0.048 | 0.142 |
| | T2. Lean-markedly-increasing trajectory | 0.004 | 0.106 | **0.181** | **0.522** |
| | T3. Lean-slightly-increasing trajectory | 0.002 | 0.047 | **0.221** | **0.580** |
| Birth weight | Low birth weight | **0.017** | **1** | 0.009 | 0 |
| | Adequate birth weight | 0 | 0.267 | **0.002** | **0.554** |
| | High birth weight | **0.007** | **0.506** | 0.049 | 0.388 |
| Maternal education | Maternal education equivalent to primary school or less | **0.046** | **0.945** | 0 | 0 |
| | Maternal education equivalent to complete secondary school or university | **0.129** | **0.945** | 0 | 0 |
| BMI | Normal range | 0 | 0.004 | **0.198** | **0.583** |
| | Pre obesity | **0.001** | **0.364** | 0.001 | 0.046 |
| | Obesity | 0.001 | 0.015 | **0.263** | **0.600** |
| Per capita family income | Low per capita family income | **0.168** | **0.803** | 0 | 0 |
| | Middle per capita family income | **0.008** | **0.784** | 0.001 | 0.008 |
| | High per capita family income | **0.106** | **0.794** | 0.001 | 0.001 |
| Race | White race | **0.072** | **0.952** | 0.001 | 0.001 |
| | Brown race | **0.041** | **0.947** | 0.004 | 0.011 |
| | Black race | **0.064** | **0.911** | 0.0021 | 0.034 |
| Education | Secondary education or less | **0.174** | **0.789** | 0 | 0 |
| | Complete university education | **0.133** | **0.789** | 0 | 0 |
